# Chromatin unfolding via loops can drive clustered transposon insertion

**DOI:** 10.1016/j.bpj.2025.03.038

**Published:** 2025-04-04

**Authors:** Roshan Prizak, Aaron Gadzekpo, Lennart Hilbert

**Affiliations:** 1 Karlsruhe Institute of Technology, Institute of Biological and Chemical Systems, Eggenstein-Leopoldshafen, Germany; 2 Karlsruhe Institute of Technology, Zoological Institute, Karlsruhe, Germany

## Abstract

Transposons, DNA sequences capable of relocating within the genome, make up a significant portion of eukaryotic genomes and are often found in clusters. Within the cell nucleus, the genome is organized into chromatin, a structure with varying degrees of compaction due to three-dimensional folding. Transposon insertion or activation can lead to chromatin decompaction, increasing accessibility and potentially facilitating further nearby insertions. This positive feedback between chromatin unfolding and transposon insertion may result in transposon clustering. Here, we combine bioinformatics with polymer modeling to explore possible mechanisms and conditions that promote clustered transposon insertions. Our analysis of human cell line genomic repeat data reveals extensive clustering of heterochromatic LINE-1 elements and euchromatic Alu elements. For Alu elements, this clustering correlates with increased chromatin accessibility. Both Alu and LINE-1 deviate in their sequence-inherent flexibility from the overall genome, with above-average flexibility for Alu and below-average flexibility for most LINE-1 sequences. Flexibility was highest in young transposons, so that young Alu and LINE-1 exceed overall genome flexibility. We developed an according polymer model of transposon insertion, consisting of a self-attracting chromatin domain. Transposon insertions locally disrupt self-attraction, leading to unfolding of the domain as more transposons are inserted. In simulations where transposons are inserted adjacent to existing ones, we observed gradual unfolding through loop extensions from a folded core. Including transposases as explicit particles, our model shows that adjacent transposon insertion occurs when densely packed chromatin excludes transposases or when insertion rates exceed the thermal equilibration rate of polymer configurations. We conclude that 1) dense chromatin packing that hinders transposase access as well as 2) a local loss of compaction upon transposon insertion favor clustered transposon insertion via loop formation. This biophysical mechanism of clustered insertion site preference would act in combination with selective pressures shaping transposon distribution over evolutionary timescales.

## Significance

A large part of the genome is composed of repetitive sequences, so-called transposons. Transposons are involved in important processes, such as early embryonic development or control over which genes are used by the cell. Transposons frequently occur in clusters, where many similar sequence motifs are grouped together. Recent studies suggest that the insertion of transposons can result in local unfolding of the genome, favoring insertion of yet more transposons. Our work simulates a simplified region of the genome and transposases, which are the molecules that insert transposons into the genome. Surprisingly, fast-acting transposases that are excluded by compacted chromatin favor the formation of loops that contain most of the inserted transposons, providing a potential explanation for clustered insertion of transposons.

## Introduction

Transposons, also called transposable elements, are sequences of DNA that are capable of inserting themselves into new locations in the genome. Transposon insertion often disrupts the function of existing DNA sequences, such as regulatory sequences and genes, and can compromise genome stability (1). These disruptions can lead to disease (2), including carcinogenesis and tumor progression (3,4,5). Accordingly, organisms have developed mechanisms to prevent transposition and remove or repress transposons (6,7). However, due to high transposition rates, transposons also contribute to genotypic and phenotypic variation (8,9,10). As major drivers of genome evolution, they often get retained in the genome and acquire new functions. For instance, they contribute to gene regulation (11), replication (1), and development (12). Transposons make up a surprisingly large fraction of eukaryotic genomes—about 46% of the human genome, and an astonishing 85% of the maize genome, highlighting their ubiquity.

Bioinformatic analysis of reference genomes reveals that transposons are not uniformly distributed throughout the genome, but in clusters. This transposon distribution is shaped by a combination of insertion processes and evolutionary changes in DNA sequence, but the relative contributions of these processes is still under debate (13). De novo insertions cluster on the 10 kb to 1 Mb scale, and often show a preference for regions containing active genes and previously inserted transposons of the same kind (13,14,15). Transposon insertion is mediated by biological molecules such as transposases and ribonucleoprotein complexes, which have to navigate the nuclear space, shaped in part by the three-dimensional (3D) organization of chromatin (16,17,18,19,20). The transposase accessibility of chromatin can be assessed by the according Assay for Transposase-Accessible Chromatin (ATAC) (21). Combining this method with sequencing (ATAC-seq) reveals differences in accessibility at the level of the chromatin fiber and nucleosome arrays (21). Combination with fluorescent tagging and imaging (ATAC-see) suggests that also at scales of 100 nm and above, decompacted chromatin is more accessible to transposases (22). Differential accessibility for transposition might result from steric restriction (16,18,20,23) or obstruction of the formation of relevant macromolecular assemblies by the surrounding chromatin meshwork (24). Accordingly, differences in large-scale chromatin organization seem to play a role in the nonuniform insertion of transposons.

Recent studies have pointed out how transposon activity could, in turn, shape 3D chromatin organization. LINE-1 and Alu repeats, for example, have been implicated in the spatial separation of the genome into the A and the B compartment (25). Transposons can also serve as contact domain boundaries, as illustrated by the MERVL family in the early mouse embryo (26), the HERV-H family in human pluripotent stem cells (27), and the MIR family in CD4^+^ T cells (28). Transposon insertion can also perturb chromatin organization, for instance, by disrupting sequence elements that set up chromatin organization, and lead to increase in chromatin accessibility (12,26). Transposon activation and transcription can also induce chromatin unfolding and relocation of transcribed regions into the active nuclear compartment, away from inactive chromatin (29,30,31,32,33,34,35,36,37). Thus, both transposon insertion and transposon activity can trigger chromatin unfolding, potentially leading to chromatin configurations that favor transposase accessibility.

These observations suggest a possible feedback between transposition-mediated chromatin unfolding and further propensity for transposon insertion. While such a feedback can be expected to favor clustered insertion, it is still poorly understood by what exact process and under which conditions this feedback might take effect. In this study, we combined bioinformatics analysis and polymer simulations to assess how the positive feedback between transposon insertion and chromatin unfolding affects the distribution of newly inserted transposons. We find that unclustered insertion of transposons leads to a sharp unfolding of chromatin, while clustered insertion leads to a gradual unfolding in the form of loops. Furthermore, we find that transposases that are excluded from compacted chromatin and faster transposition favor this unfolding in the form of loops, resulting in clustered transposon insertion. We thus propose a biophysical mechanism that can contribute to clustered integration site preference and would act in combination with selective pressure, shaping transposon distribution over evolutionary timescales.

## Materials and methods

### Bioinformatic analysis

Transposon positions for the hg19 genome build were obtained from the RepeatMasker (38) track provided through the UCSC genome browser (39,40). Transposon subfamilies were selected by repName annotation. For comparison, previously published de novo L1 integrations into HeLa-S3 cells (13) were evaluated, we therefore excluded the X-chromosome from further analysis. Centers of transposons were used to calculate pairwise distances and for cluster detection with DBSCAN. For each annotated transposon, a reference element with the same length was placed at a random position chosen from a uniform distribution spanning the genomic range covered by transposons. The probability density for pairwise distances from continuously and uniformly distributed points has the expression $p=2\left(L-x\right)/L^{2}$, were *p* is the probability to find a pairwise distance x given a genomic range L (details and derivation can be found in a python script on GitHub, link see below). Histograms of pairwise distances between transposons were compared with the theoretical prediction for a uniform placement. Logarithmic scaling for bin sizes and axes was used, as deviations from a uniform placement mainly occurred for small pairwise distances. DBSCAN implemented in scikit-learn (version 1.1.2) was used with a maximum sample distance of 10,000 nucleotides (capturing the peak of pairwise distances between L1ME1 elements) and a minimum sample number of 2. For open chromatin regions, dataset ENCSR236YNV from HeLa-S3 cells was downloaded from ENCODE (41,42,43). To ensure high confidence in annotated regions, a subset of peaks found in both DNaseI and FAIRE assays or with a combined *p* value of less than 0.01 were used. The fraction of transposons or reference elements on each chromosome found in clusters as well as the fraction overlapping open chromatin were visualized in boxplots, complemented with exemplary, annotated chromatin sections.

To evaluate whether transposons differ in their sequence-encoded mechanical properties from other regions of DNA, we used the TRX scale (44). The TRX scale relates NMR data of the BI <–> BII equilibrium of the phosphate link of DNA to the local DNA flexibility. TRX scores quantify the percentage frequency that a given dinucleotide and its complement are detected in the BII conformation. High TRX scores correlate with high flexibility expressed in Twist, Roll, and basepair displacement (X-disp). DNA sequences with strong binding to nucleosomes showed alternating patterns of above and below average TRX scores, with a periodicity of ≈10 basepairs (44). Accordingly, the TRX score provides a DNA sequence-inherent interaction strength between DNA and its binding proteins. We calculated a genome-wide, averaged TRX score as well as averaged scores for transposon families, uniformly distributed reference elements, genes, and A/B compartments in HeLa cells. Annotation for 99,524 gene features (excluding the X-chromosome) was obtained from the wgEncodeGencodeBasicV19 set provided through the UCSC genome browser. Annotation for A/B compartments has previously been generated with a machine learning method called SNIPER using Hi-C data (45). The annotation for intervals of 100 kb on autosomes of HeLa cells was downloaded from http://genome.compbio.cs.cmu.edu:8008/∼kxiong/data/sniper/annotations/. To investigate whether TRX scores correlate with evolutionary age of transposons, we used a chronological ordering of transposon subfamilies based on the level of transposon defragmentation. A table of all ordered transposon subfamilies was downloaded from the supplemental information of (46). We used 360 subfamilies from this table that could be matched by name to the hg19 transposon annotation used in our other analyses. For those subfamilies, we also carried out the clustering analysis and correlation with open chromatin regions as described above. A table containing TRX scores and overlap with open chromatin can be found in the supporting material. When comparing distributions, *p* values were calculated using a Mann-Whitney U rank test for two-sided (unequal) distributions (notches in boxplots indicate 95% bootstrap confidence intervals). The entire evaluation script with additional details, figures and analysis can be found on GitHub (https://github.com/aaron-gad/Transposon_Clustering).

### Simulation of a chromatin domain and transposons

We model a 300-kb region of chromatin as a polymer chain with $N_{\text{tot}}=100$ monomers. Each monomer represents a 3-kb chromatin region, and is assumed to have an effective diameter of σ=30nm. This implies a chromatin packing of 100bp/nm, which compares well with a previous estimate of 90−130bp/nm (47). In addition to the monomers that represent the chromatin chain, the simulation can also contain particles of variable diameter, which represent transposon molecules. We simulate the spatial dynamics of the system in the molecular dynamics simulator LAMMPS (48). Particles interact via phenomenological force fields (potentials) and follow Newton’s laws of motion. At the core of the simulation is a standard Velocity-Verlet algorithm that solves the underlying Langevin equations describing the system dynamics.

The simulation parameters are assigned effective values that include the influence of typical chromatin-associated proteins and RNAs based on previous work. The chromatin polymer is held together by harmonic springs between adjacent pairs of monomers, and the polymer’s bending rigidity is modeled by a Kratky-Porod potential. For the chromatin chain, we assume a persistence length of 3 monomers, or 90nm, in line with experimental estimates of 50−200nm (49) and 66−134nm (50). To model pure (steric) repulsion between any pair of atoms, we consider the Weeks-Chandler-Andersen potential, which is a shifted version of the Lennard-Jones potential with a cutoff at the zero-crossing point. To model steric repulsion, this cutoff distance is typically set to be the mean diameter of the two interacting atoms. As all the chromatin monomers have the same diameter *σ*, for all monomer pairs, the cutoff distance is set as $r_{\text{cut}}=2^{1/6}\sigma$.

Chromatin monomers can be of two types—native or transposed. Only native monomers attract each other when they are in close proximity. Transposon insertion converts a native monomer to a transposed monomer, which does not exhibit attraction with other monomers. The chromatin monomers in their unmodified (“native”) state are assigned an interaction energy of 1.5 $k_{B}T$ between monomers, in line with parameters used in previous chromatin polymer simulations (49,51). The attractive interactions are modeled by using a shifted and truncated Lennard-Jones potential with a larger cutoff distance of 2.5σ. Hence, when a pair of native monomers are within a distance between $2^{1/6}\sigma$ and 2.5σ, they experience an attraction.

Transposases have a size $s_{T}\sigma$, so the cutoff distance for the repulsive Weeks-Chandler-Andersen potential between any pair of transposases is $2^{1/6}s_{T}\sigma$. For a monomer-transposase pair, we set the cutoff distance to be $2^{1/6}s_{T}\sigma$, a value smaller than the mean diameter of a monomer-transposase pair. This allows the transposase molecule to come closer to a monomer molecule than another monomer molecule can. Moreover, the cutoff distance is smaller for smaller transposases, allowing them to come closer to monomers than larger transposases. This implementation uses different transposon “sizes” to represent different strengths of effective exclusion of transposases from chromatin.

### Prescribed and emergent transposon placement

Transposon insertions are implemented differently in two different versions of the model. In the “prescribed adjacency model,” the placement adjacency parameter, α∈[0,1], is fixed at the beginning of each simulation run to directly prescribe the extent of clustered transposon insertion. In every insertion phase of the simulation run, either an adjacent transposon is placed with probability *α* or a uniformly distributed transposon is placed with a probability 1−α. At α=0, we generate polymers for which all insertion positions are randomly chosen from all available positions and, at α=1, we generate polymers with insertion positions picked only from available positions that are adjacent to existing transposons.

In the “emergent adjacency model,” the interaction of transposases with chromatin and the resulting transposon insertion are modeled explicitly. To this end, during every insertion phase of the simulation, all native monomers that have a transposase molecule within a distance of $\left(1+s_{T}\right)\sigma$ are converted to a transposon monomer. The involved transposase molecules are then removed from the simulation and an identical number of new transposase molecules is added at the boundary of the simulation volume to conserve the total number of transposases. To quantify the extent of placement adjacency that emerges from this simulation of the insertion process, we perform a maximum likelihood estimation of the parameter value $\alpha_{\text{mle}}$ that best explains the first 50 insertions.

### Simulation procedure and evaluation of outcomes

Each simulation run in LAMMPS starts with an initial polymer with no transposons and consists of an initial “soft” phase, an equilibration phase, and finally the main simulation block in which transposons are sequentially inserted into the chromatin polymer. In the model without transposases, the main simulation block contains $100,000\left(N_{\text{tot}}+1\right)$ timesteps, and insertion is executed every 100,000 timesteps. In the model with transposases, insertion is attempted every $1000/k_{T}$ timesteps, leading to 100−10,000 timesteps per simulation segment. In between simulation segments, every native monomer that has a transposase within a distance of $\sigma \left(1+s_{T}\right)/2$ is converted to a transposed monomer. The transposase is removed and a new transposase is added to the simulation volume boundary. All simulation segments are of equal duration, containing enough time steps to allow the polymer to equilibrate between consecutive insertions. For each simulation run and number of inserted transposons $N_{\text{tr}}$, we quantify the overall level of compaction by computing the polymer’s radius of gyration, $R_{g}$.

When combinations of transposition rate $k_{T}$ and transposase size $s_{T}$ are scanned, for each $\left(k_{T},s_{T}\right)$ combination, 25 simulation runs are executed with different random seeds and averaged.

For full model details, see supporting material, code: https://doi.org/10.5281/zenodo.14788016.

## Results

### Heterochromatic compaction as well as local unfolding correlate with transposon clustering

**Figure 1:**
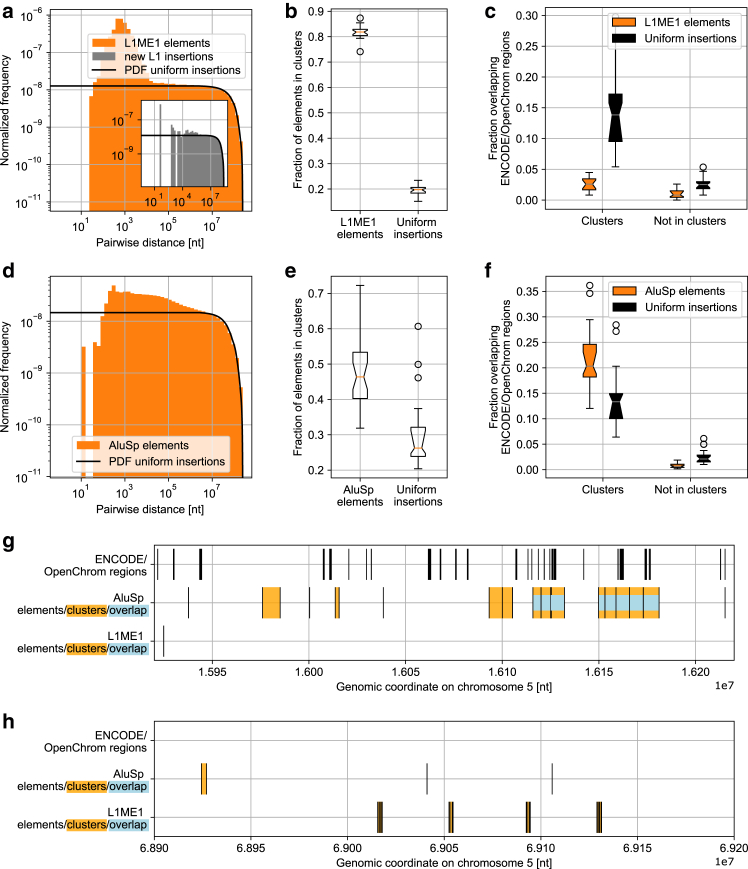
Clustering analysis of representative transposons, L1ME1 and AluSp. (*a*) Normalized histogram of pairwise center-center distances in nucleotides (nt) between 31,377 L1ME1 elements in the hg19 human reference genome build compared with the probability density resulting from uniformly distributed insertions. Inset shows de novo integrations in HeLa-S3 cells (13). Y-chromosome excluded for comparability. (*b*) Fraction of L1ME1 or uniformly distributed elements in clusters detected by DBSCAN (max. dist. = 10,000 nt, min. points = 2). Boxplot shows spread between chromosomes (*p* < $10^{-8}$). (*c*) Fraction of L1ME1 or uniformly distributed elements of same length overlapping open chromatin regions from ENCODE/OpenChrom dataset in HeLa-S3 cells. Boxplot shows spread between chromosomes (in clusters: *p* < $10^{-6}$, not in clusters: *p* < $10^{-6}$). (*d*) Pairwise distances between 48,743 AluSp elements compared with probability density from uniformly distributed insertions. (*e*) Fraction of AluSp or uniformly distributed elements in clusters detected by DBSCAN (*p* < $10^{-5}$). (*f*) Fraction of AluSp or uniformly distributed elements of same length overlapping open chromatin regions in HeLa-S3 cells (in clusters: *p* < $10^{-4}$, not in clusters: *p* < $10^{-5}$). (*g*) A 300,000 nt section on chromosome 1 containing a high number of clustered AluSp elements but few L1ME1 elements. Note the overlap with regions of open chromatin (*blue color*). Genomic coordinates given in nt. (*h*) A 300,000 nt section on chromosome 5, depleted in both AluSp and open chromatin regions, but with a high number of clustered L1ME1 elements.

To assess the applicability of the core assumptions before the actual construction of a simulation model, we analyzed the distribution of representative LINE-1 and Alu repeats. In human cells, LINE-1 repeats occur within heterochromatin, Alu repeats in euchromatin (25). Analysis of the genomic distribution of two according subfamilies, L1ME1 and AluSp (each containing transposons with similar sequence), reveals the expected clustered placement: in both cases, short pairwise distances ($<10^{5}$ basepairs) occur more frequently than expected from uniformly distributed elements (Fig. 1, *a* and *d*). A similar increase in short pairwise distances was also found for de novo insertions of LINE-1 elements, indicating that the clustering already occurs during transposition (13) (Fig. 1 *a*, *inset*). Note that the cluster sizes (<100 kb) are below the typical sizes of endogenous euchromatin and heterochromatin domains (several Mb), so that several transposon clusters can occur within a given epigenetic domain.

The pairwise distance distribution suggests stronger clustering for L1ME1 (Fig. 1, *a* and *d*). Indeed, a direct quantification using DBSCAN shows that L1ME1 more frequently occurs in clusters than AluSp (Fig. 1, *b* and *e*). This effect is robust against changes in the clustering parameters, and occurs despite the higher number of AluSp elements (48,743 vs. 31,377), which would favor clustering of AluSp when assuming a uniform distribution pattern.

The algorithm calls clusters using center coordinates of transposons, meaning that the lengths (a mean of 481 and 289 nucleotides was calculated for L1ME1 and AluSp, respectively) only affects the total length of clusters. With a mean length of 3.6 kb and maximum length of 32 kb, clusters of L1ME1 are on average smaller than those of AluSp elements (8.2 and 85 kb). Mean and maximum cluster lengths from uniform insertions in both cases are approximately 6 and 30 kb. Absolute values can change with the parameters used in DBSCAN but relative differences are retained.

Thus, L1ME1, which is confined to heterochromatic, more compacted genome regions, exhibits stronger clustering than AluSp, which occurs in euchromatic, less-compacted regions (52). Accordingly, chromatin compaction should be considered as one crucial aspect of our transposon insertion simulations.

Besides general chromatin compaction encoded by epigenetic state, we also assessed whether local accessibility is correlated with transposon clustering. Based on data from a human cell line, L1ME1 transposons are depleted in open chromatin regions, independent of clustered or isolated occurrence (Fig. 1 *c*). Differently, clusters of AluSp are preferentially found in open chromatin (Fig. 1 *f*). Isolated AluSp are not enriched in open chromatin (Fig. 1 *f*). In both cases, we used transposon positions with uniformly distributed insertions of the same lengths as a base line. When we removed influence from the lengths of the elements and regions, instead using center coordinates, the same results were obtained. These results imply that the relation between clustered transposon insertion and chromatin opening can be detected as long as no additional repression by heterochromatic silencing has occurred subsequently to the initial transposon insertion.

The differences between AluSp and L1ME1 are also apparent when visualizing example genome sections. Clusters of AluSp are often wider and overlap with open chromatin regions, while L1ME1 clusters are typically shorter and depleted in regions annotated for open chromatin. L1ME1 elements are rarely found outside of clusters and cover regions with few AluSp elements (Fig. 1, *g* and *h*).

To ensure that our findings are not limited to two special cases, we repeated the analysis with more LINE and Alu subfamilies (Fig. S1). Pairwise distance distributions and fractions of elements in clusters show characteristics for LINE and Alu similar to the two subfamilies discussed here. Compared with uniform insertions, overlap of clusters with open chromatin can be reduced (L1ME2, L1MB3, L2), similar (L2b), or increased (AluJr, AluSq2).

### Increased DNA flexibility is encoded by young transposon subfamilies

**Figure 2:**
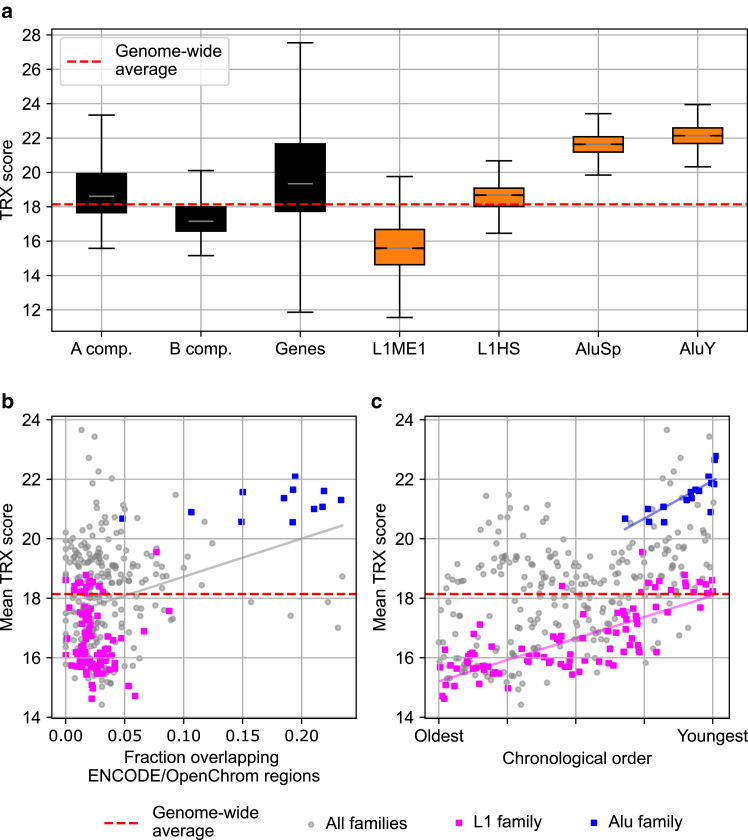
Transposon flexibility quantified by TRX scores. The TRX scale quantifies sequence-inherent flexibility; high values correspond to high flexibility (44). (*a*) Boxplots contain the average TRX scores for all L1ME1, L1HS, AluSp, or AluY elements. Comparison with averaged TRX scores of control elements yielded *p* values smaller than $10^{-57}$ (Fig. S2). For reference, TRX scores averaged genome-wide, in A/B compartments of HeLa cells (45), and in genes were calculated. (*b*) TRX scores averaged for all members of one subfamily (e.g., AluSp or L1ME1) were plotted against the fraction of transposon clusters in each subfamily that overlaps open chromatin. Open chromatin annotation obtained from the ENCODE/OpenChrom dataset for HeLa-S3 cells. A total of 321 subfamilies that had at least 10 clusters on at least one chromosome were analyzed. Linear fit with $R^{2}=0.09,p_{slope}=3.0\times 10^{-8}$. (*c*) Chronological ordering of 360 transposon families (46) reveals that younger L1 and Alu subfamilies have increased average TRX scores. Linear fit with $R^{2}=0.59,p_{\mathrm{slope}}=4.5\times 10^{-19}$ and $R^{2}=0.61,p_{\mathrm{slope}}=3.7\times 10^{-4}$ for L1 and Alu, respectively.

Because transposon clustering and chromatin accessibility coincided only for some of the above transposons, we wondered how and when transposons contribute to accessibility. One explanation, as discussed in the introduction, is an increase in accessibility upon transposon insertion and activation. Additionally, the very DNA sequence of transposons could be imagined as a cause of increased chromatin accessibility. To assess sequence-inherent flexibility, we calculated TRX scores, which quantify the flexibility of double-stranded DNA based on its sequence, using NMR measurements for specific dinucleotides (44). To obtain a general point of reference, we generated an average TRX score for the entire genome (Fig. 2 *a*). Relative to this genome-wide reference, TRX scores for all genes and all A compartment domains were slightly higher, and B compartment domains were slightly lower (Fig. 2 *a*). To test the applicability of this approach for transposons, we compared the averaged TRX scores of all transposons in a given subfamily to the TRX scores of uniformly placed reference elements. Indeed, TRX scores of reference elements were centered around the genome-wide average, whereas transposons deviated markedly from the average (Fig. S2). Having confirmed the sensitivity of our approach, we again compared members of the heterochromatic LINE-1 family with members of the euchromatic Alu family. L1ME1 had a TRX score below that of the overall genome as well as the typically heterochromatic B compartment (Fig. 2 *a*). In contrast, elements of the currently active, evolutionarily young (46,53) L1HS subfamily had TRX scores exceeding the B compartment as well as the overall genome, representing an example of increased flexibility of a heterochromatic transposon (Fig. 2 *a*). Strongly increased TRX scores were obtained for AluSp and the young, active (46,53,54) AluY subfamily (Fig. 2 *a*).

### Flexible transposon subfamilies are associated with open chromatin

**Figure 3:**
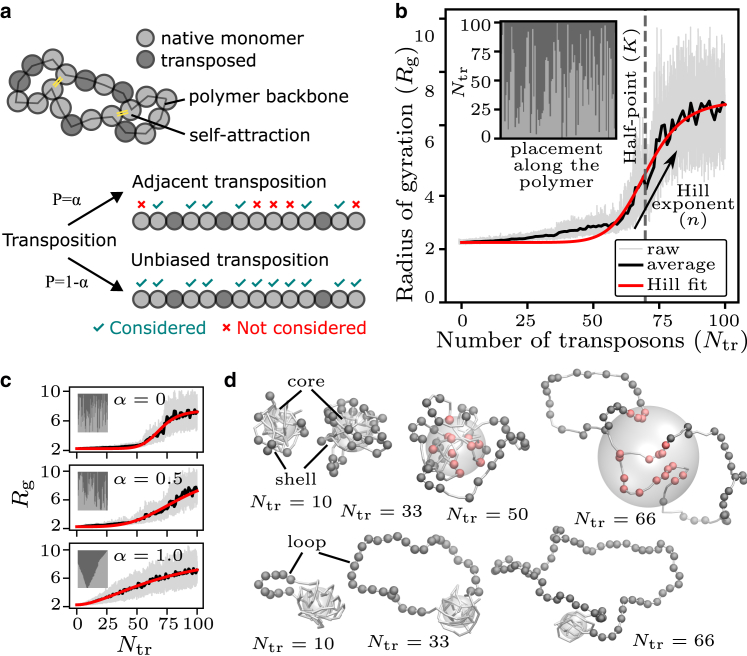
Adjacent transposon insertion converts a sharp unfolding transition into gradual loop formation. (*a*) Schematic of the model chromatin polymer consisting of native monomers, which attract each other (*yellow*), and transposed monomers, which do not exhibit attraction. The placement adjacency (*α*) value controls the probability of a new transposition occurring next to an already transposed monomer. (*b*) The radius of gyration ($R_{g}$) of a chromatin polymer of fixed length ($N_{\text{tot}}=100$) increases as transposons accumulate. Transposons accumulate one at a time, starting from a polymer with no transposons. The insertion history, referring to the sequence of positions on the linear polymer where transposons are inserted, is depicted in the inset. We fit a Hill curve to $R_{g}\left(N_{\text{tr}}\right)$ to capture the sharpness of unfolding and the transition point. (*c*) α=0 leads to sharp unfolding, nonzero values of *α* lead to gradual unfolding. (*d*) Example snapshots (*top row*: α=0; *bottom row*: α=1) from the polymer simulations at different numbers of transposons ($N_{\text{tr}}$). Native monomers are shown as a white backbone. A semitransparent white sphere with a radius equal to 40% of the bounding sphere for native monomers is shown to depict the polymer core. Transposed monomers are shown either in dark gray (if they are outside the semitransparent sphere) or in pink (if they are inside the semitransparent sphere). For (*b*) and (*c*), which serve for illustrative purpose only, an interaction strength of 2.0 $k_{B}T$ was used.

**Figure 4:**
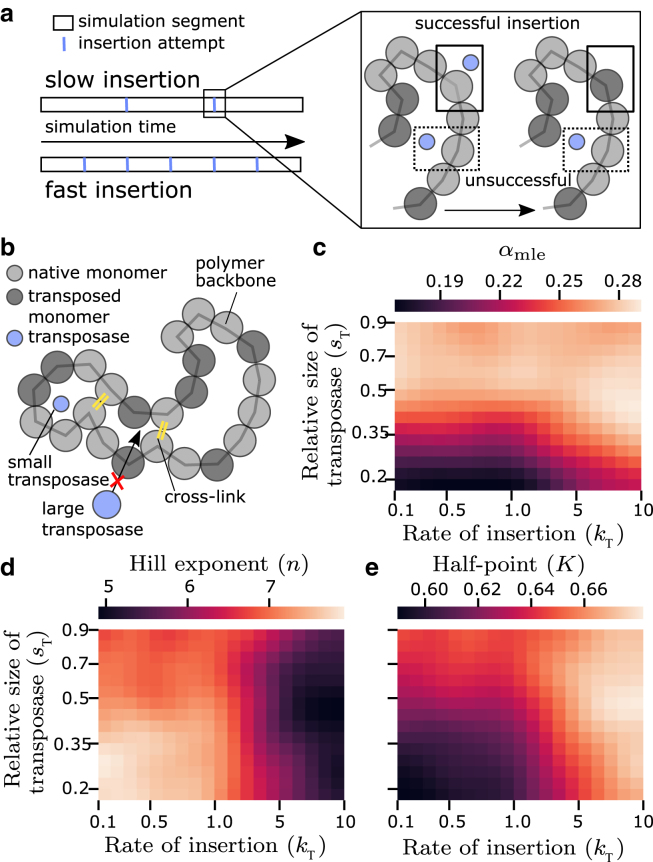
Large transposases and fast insertion result in gradual unfolding and adjacent transposon insertion. (*a*) A simulation run is composed of multiple simulation segments interspersed by transposon insertion attempts. In each simulation segment, the chromatin polymer and other molecules in the system move according to the potentials they experience. In each insertion attempt, native monomers close to a transposase are converted to a transposed monomer. The transposon insertion rate is implemented by changing the number of iteration steps in the simulation segments in between consecutive insertion attempts. (*b*) Schematic of the polymer model with transposon insertion mediated by diffusing transposases. Differential accessibility of chromatin is considered via a size-based exclusion, which restricts access to compacted polymer regions. (*c*) The maximum-likelihood estimate $\alpha_{\text{mle}}$ for simulation runs for different values for the rate of insertion $k_{T}$ and relative size of transposase $s_{T}$. (*d* and *e*) Hill exponent *n* and half-point *K* of the Hill curve fitted to $R_{g}\left(N_{\text{tr}}\right)$ for different values of $k_{T}$ and $s_{T}$.

**Figure 5:**
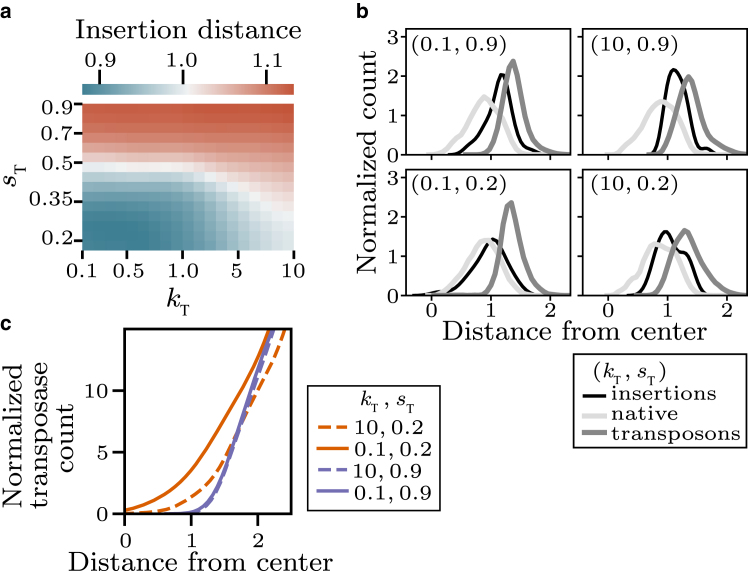
Clustered insertion is driven by exclusion of transposases from the polymer core. (*a*) Average insertion distance (first 30 insertions, relative to polymer center, normalized by $R_{g}$) for different effective transposase size $s_{T}$ and insertion rate $k_{T}$. (b) Distance distribution of native monomers, transposed monomers, and insertion events (first 30 insertions) for 4 different sets of $s_{T}$ and $k_{T}$. (c) Distance distribution of transposases for four different sets of $s_{T}$ and $k_{T}$.

**Figure 6:**
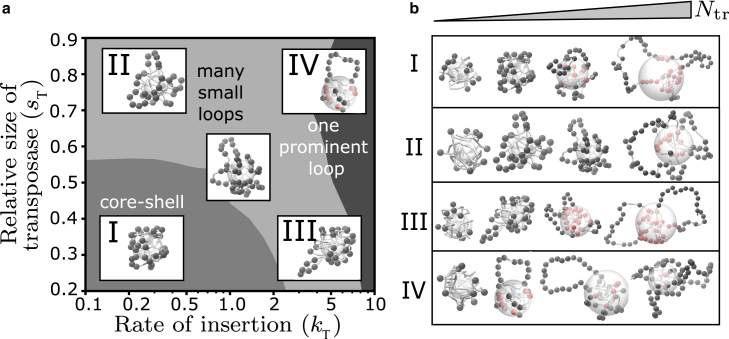
Different scenarios of transposon insertion and polymer unfolding. (*a*) Modulation of effective transposase size and insertion rate leads to four different scenarios of transposon-induced unfolding. In scenario I, small transposases and slow insertion lead to unclustered insertion and polymer configurations consisting of a compact core of native monomers and a shell of transposed monomers around it. Scenarios II and III exhibit unfolding via the formation of several small loops that occur as a result of clustered insertion—either because of size-based exclusion of large transposases or fast insertion on the outer shell. Scenario IV typically exhibits one prominent loop of clustered transposons. (*b*) Representative snapshots of polymer simulations at increasing numbers of transposons in the four scenarios (left to right, $N_{\text{tr}}\approx 10,33,50,66$).

From the TRX scores of the four example transposons, we hypothesized that evolutionarily younger transposons are more flexible, potentially contributing to increased chromatin accessibility. An evaluation of flexibility (TRX score) and accessibility (overlap of transposon clusters with accessible chromatin regions) over a total of 321 transposon subfamilies revealed a broad distribution, which nevertheless produced a statistically significant correlation between accessibility and flexibility ($R^{2}=0.09,p_{\mathrm{slope}}=3.0\times 10^{-8}$ for linear fit, Fig. 2 *b*). Further supporting this interpretation, the Alu family is mostly found in the region of high flexibility and accessibility, whereas the L1 family is mostly found in the region of low flexibility and accessibility (Fig. 2 *b*). A scatter plot labeling all families can be found in Fig. S3 *b* of the supporting material. Given the considerable variability even within the Alu and L1 family, we assessed evolutionary age (chronological order based on fragmentation due to insertions) as an additional ordering variable (46). Indeed, considering 360 subfamilies, a decrease of flexibility with increasing evolutionary age was visually apparent (Fig. 2 *b*). This trend was statistically confirmed for the subfamilies of the L1 and Alu families ($R^{2}=0.59,p_{\mathrm{slope}}=4.5\times 10^{-19}$ and $R^{2}=0.61,p_{\mathrm{slope}}=3.7\times 10^{-4}$ for linear fit, respectively). The flexibility of the Alu family was generally higher than the whole-genome average (Fig. 2 *c*). For L1 transposons, particularly the youngest L1 subfamilies exceed the genome-wide average (Fig. 2 *c*). A decrease in flexibility with increasing age occurs also for other families, such as EVR1, but is absent for others, such as hAT-Tip100 (Figure 1, Figure 2, Figure 3, Figure 4, Figure 5, Figure 6*a*; Table S1). Taken together, we find that the sequence composition of transposons can, indeed, inherently encode differences in DNA flexibility. Such differences are known to compromise the protein-binding capability, especially nucleosome binding, required for chromatin compaction (44). In our analysis, high sequence-encoded flexibility of a given transposon subfamily coincides with increased overlap of transposon clusters with open chromatin, strongly supporting this hypothesized relationship. Lastly, high sequence-encoded flexibility and association with open chromatin are properties of young transposons, which are less pronounced with increasing evolutionary age.

### Clustered transposon insertion induces gradual chromatin unfolding via loops

Previous work (see introduction) as well as our TRX scale analysis indicate that de novo integration of transposons can locally decompact chromatin and increase accessibility. To understand how an increasing number of transposons that are integrated would affect the overall configuration of a larger chromatin domain, we carried out molecular dynamics simulations of a 300-kb chromatin domain undergoing successive transposon insertion. The simulated domain initially consists only of native monomers, which attract each other when they are in close proximity, resulting in a compactly folded configuration (Fig. 3 *a*). Transposon integration is implemented as a one-by-one conversion of native monomers into “transposed” monomers, which are no longer attracted to other chromatin monomers. Chromatin polymers with few transposons still adopt a compact configuration facilitated by attraction between the native monomers. With further transposition, the polymer unfolds into an open configuration. As the polymer’s radius of gyration $R_{g}$ represents the effective size of the polymer, this unfolding is quantified by the observed increase in $R_{g}$ with the number of transposed monomers $N_{\text{tr}}$ (Fig. 3 *b*). We fit a Hill curve to $R_{g}\left(N_{\text{tr}}\right)$ to capture the sharpness of unfolding in terms of the Hill exponent *n* and the transition point in terms of the half-value *K* (Fig. 3 *b*; see supporting material for details). A larger Hill exponent characterizes sharper unfolding, and a larger half-point characterizes a transition at a higher number of transposons.

We can now use our simulations to assess how different degrees of clustered transposon insertion might affect chromatin unfolding. To initially control the extent of clustering directly, we introduce the placement adjacency value α∈[0,1] as a control parameter. During the one-by-one insertion of transposons, the value of *α* controls the choice of the next native monomer that gets converted into a transposed monomer. For increasing values of *α*, it is increasingly likely that a native monomer located directly next to an already transposed monomer is chosen for the next conversion into a transposed monomer. The influence of varying *α* is also reflected in the insertion histories: we obtain insertion histories ranging from no placement adjacency (α=0) and hence no clustering, to maximal placement adjacency (α=1) resulting in complete clustering (*insets*, Fig. 3 *c*). Placement adjacency *α* strongly affects the transition—while α=0 results in a sharp, switch-like unfolding, transposon clustering at α=1 results in a gradual unfolding. In other words, when transposons are preferentially inserted in transposon-adjacent positions, transposon clusters emerge and the polymer unfolding becomes more gradual (Fig. 3 *c*).

The influence of placement adjacency on polymer unfolding upon $N_{\text{tr}}$ can be understood by visualizing the 3D polymer configurations (Fig. 3 *d*). As the first transposons get inserted, they form a shell on the outer layer of the compact polymer core. For low *α*, as transposons accumulate further, the shell grows, but no unfolding occurs as long as the polymer core is intact (core-shell configuration). The core starts to open up around the transition point, leading to a sharp unfolding. On the other hand, clustered transposon insertion at large *α* leads to a transposon cluster looping away from the compact polymer core (loop configuration). This loop increases in length with further transposition, leading to a gradual increase in $R_{g}$. Taken together, we find that while unclustered transposon insertion leads to sharp unfolding via the core-shell polymer configuration, clustered transposon insertion leads to gradual unfolding via loop formation.

### Transposase exclusion from chromatin and rapid transposon insertion favor clustered insertion

The observation that clustered transposon insertion leads to gradual unfolding begs the question of how such an insertion history might emerge. Transposons are preferentially inserted in less densely packed chromatin (see introduction), suggesting that clustered insertion might arise from chromatin packing differences. To further investigate this possible mechanism, we now extend our simulations to explicitly treat transposase molecules (Fig. 4, *a* and *b*). In this extended model, insertions occur when a transposase molecule comes close to a native, not transposed chromatin monomer. The insertion history therefore depends on the access of transposase to different parts of the polymer chain and emerges out of the transposase-chromatin interactions in 3D space. We assess the impact of two properties of the transposases. First, we consider the exclusion of transposase molecules from dense chromatin, which we implement by assigning different values for the effective transposase size, $s_{T}$ (Fig. 4 *b*). A larger effective size here represents stronger effective exclusion. Exclusion of macromolecules from densely folded chromatin has been theoretically discussed (16,18) and experimentally shown (17,23). Our simulations are agnostic to the underlying cause for this exclusion, seeing that steric exclusion as well as specific repulsive molecular interactions might be responsible. Effective pore sizes of 16–20 nm were determined for highly compacted chromatin (17,55), matching well with the diameter of proteins ranging from 5 to 20 nm (18), implying volume-based exclusion of large proteins from highly compacted chromatin as one possible mechanism besides specific molecular interactions. Second, we vary the rate of transposition, $k_{T}$ (Fig. 4 *a*). While the spontaneous rates of transposition are low (smaller than 1 per genome per generation), transposition bursts with high rates of transposition (5–10 transpositions per genome per generation) have been shown to occur in stress-related conditions in various species (10,56,57). We consider a wide range of transposition rates, with low rates accounting for transpositions slower than, and high rates accounting for transpositions much faster than the chromatin polymer equilibration time.

To characterize the polymer unfolding profile, we fit Hill curves to $R_{g}\left(N_{\text{tr}}\right)$ traces for different effective transposase sizes and insertion rates (Fig. 4, *d* and *e*). For large transposases or fast insertion, the resulting Hill exponents are small, pointing to gradual unfolding as observed previously for large *α*. On the other hand, for small transposases or slow insertion, the resulting Hill exponents are larger, pointing to sharp unfolding as observed previously for small *α*. In the basic model, we saw that *α* and the resulting transposon clustering determined the unfolding profile. To check whether the differences in Hill exponents result from differences in transposon insertion bias, for each insertion history we perform a maximum likelihood estimation of the underlying placement adjacency $\alpha_{\text{mle}}$ (Fig. 4 *c*, see materials and methods and supporting material for details). We find that smaller transposases and slower insertion result in fewer adjacent insertions, consistent with the sharp unfolding and large Hill exponents. Thus, both exclusion from compactly folded chromatin (larger transposases) and quicker insertion result in more adjacent insertions, in line with gradual unfolding and small Hill exponents (Fig. 4, *d* and *e*).

### Transposase exclusion from compacted chromatin and high insertion rates favor transposition in the polymer shell

Based on the observation of a core of native monomers in some of our simulations, we hypothesized that the ability of transposases to access this core is related to clustered transposon insertion. Indeed, for strong exclusion (large transposases) and high insertion rates, the mean normalized insertion distance was increased (Fig. 5 *a*). A more detailed analysis confirms that fast insertion and large transposase size lead to more insertion within the shell and less insertion in the polymer core (Fig. 5 *b*). When fast insertion and large transposase size are combined, insertion is almost fully restricted to the outer shell (Fig. 5 *b*). This restriction of insertions toward the outer shell is reflected by an exclusion of transposases from the polymer core (Fig. 5 *c*).

### Clustered transposon insertion is associated with unfolding via loop formation

Taken together, our model analysis suggests four scenarios of how chromatin unfolding relates to progressive transposon invasion (Fig. 6 *a*). Scenario I, which occurs for transposases that can permeate chromatin (small transposases) and slow insertion, is characterized by polymer unfolding via the core-shell mechanism (Fig. 6 *b*). Scenarios II and III are characterized by unfolding via several small loops (Fig. 6 *b*). In scenario II, transposases that are excluded from compact chromatin (large transposases) cannot penetrate past the outer polymer shell, leading to clustered transposition. In scenario III, transposases that can permeate chromatin (small transposases) can, in principle, access the polymer core, but due to their fast transposition rate become exhausted before actually reaching the core. In scenario IV, in which transposases are excluded from compact chromatin and insert transposons quickly, typically only one prominent loop emerges (Fig. 6 *b*).

The combination of strong exclusion of transposases from compact chromatin and rapid transposon insertion that results in loops and clustered insertion corresponds most closely to a transposon invasion (10,56,57). A direct experimental test of transposon clusters being related to chromatin loops would be visualization by microscopy using fluorescence hybridization (25) or live-cell sequence-specific labeling of transposons (58). The causal role of local decompaction in an otherwise compacted chromatin context can be tested by experimentally simulating a transposon invasion (59) under conditions of global decompaction of chromatin. Global decompaction occurs spontaneously in early embryonic development (12) and in stem cells (60) or can be experimentally induced by histone deacetylase inhibitor treatment, for example, with trichostatin A.

## Discussion and conclusion

In this study, we used polymer simulations of a model chromatin domain to study how clustered transposon insertion can arise from a feedback between transposon insertion and chromatin unfolding. We found that transposases that are excluded from compact chromatin as well as fast transposon insertion favor clustered insertion. The crucial assumption for this effect is that transposon integration drives unfolding of chromatin, leading to the preferred integration of further transposons at loops that protrude from a polymer core formed from chromatin without inserted transposons. Similar to our model, in polymer models of retroviral insertion, insertion in decompacted chromatin is highly favored (20). How do these model predictions connect to our bioinformatics analysis of Alu and LINE-1 in the human genome? Alu elements occur in clusters and are associated with open chromatin; LINE-1 elements cluster even more strongly than Alu, but are not typically associated with open chromatin. These observations can be made sense of when considering our polymer simulations in combination with our analysis of DNA flexibility based on the TRX scale over different evolutionary ages. At the initial time of insertion, at young evolutionary age, most transposons are more flexible than the overall genome, potentially disrupting the “native” nucleosome binding and chromatin state. Such a local disruption and associated local decompaction of chromatin, according to our polymer model, should result in loop unfolding and clustered transposons due to further adjacent insertions. The stronger compaction of heterochromatin relative to euchromatin, according to our polymer model, also implies that transposons that integrate into heterochromatin should cluster more strongly. LINE-1 elements insert into euchromatin as well as heterochromatin, whereas Alu elements integrate into gene-rich regions, often proximal to other SINEs, that are located in euchromatin (14,58). The stronger clustering of LINE-1 elements relative to Alu elements during the time of initial insertion can therefore be attributed the heterochromatic insertion that occurs only for LINE-1. Following the initial time of insertion, with increasing evolutionary age, the already inserted transposon sequences are increasingly modified toward lower flexibility. This reduced flexibility implies also a progressive loss of the transposons’ effect of opening chromatin, while the initial, clustered placement is retained. In the case of Alu, the known subfamilies are evolutionarily younger, explaining how these transposons retain their increased DNA flexibility to the current day, as also seen in our bioinformatics analysis. In the case of LINE-1, our bioinformatics analysis correlated a higher evolutionary age with a marked loss of DNA flexibility, providing a potential explanation for the lack of association with open chromatin in the case of LINE-1. More generally, this explanation outlines a possible interplay between the biophysical mechanism described by our polymer simulations during initial integration and the evolutionary processes that act subsequently.

Our bioinformatics analysis showed that several transposon subfamilies, particularly those with high DNA flexibility, are associated with increased chromatin accessibility. In support of this relationship, a previous study found that Ds, Tol2, and MMLV transposons integrate into DNA regions of increased structural flexibility across multiple species (61). Increased chromatin accessibility is frequently connected to nucleosome remodeling and a local reduction in nucleosome occupancy of DNA (62,63). In the human genome, nucleosome placement can be affected by transposons (64). Specifically, translational movement of nucleosomes along the DNA was more strongly hindered in proximity of younger SINE/LINE elements. The affected genomic regions show increased mutation rates, potentially deactivating the transposons over evolutionary time, in line with our results based on the TRX scale. In addition to the correlation between higher sequence-encoded flexibility and association with open chromatin across transposon subfamilies, also stiffer L1 sequences might provide a locally more accessible, mechanical context. Even within generally less-accessible heterochromatin, such regions might be prone to further transposon integration. For example, the active L1HS subfamily predominantly integrated into older transposons in brain tissues (53). Since a chronological ordering of subfamilies revealed that older L1 subfamilies are much stiffer than the genome-wide average, this localized mechanical context might have evolved to provide the “protective lightning rods” that were previously proposed (53), preventing integration into other, functionally important regions of the genome. Integration into evolutionarily older transposons, which according to our analysis are inherently stiffer, has also been reported for Alu and ERV1 transposons (65). Stiff DNA regions, depleted of nucleosomes, have also been found upstream of transcription start sites (66), hinting at a broad link between DNA flexibility, nucleosome occupancy, and genome regulation. Considering this complex role of DNA-encoded stiffness in integration site preference, it is important to point out that a full interpretation of TRX scores should address also periodic changes in the TRX score at a frequency of 10 nucleotides (44). Also, more recent work has confirmed that the binding of nucleosomes to DNA is correlated with distinct patterns of high and low DNA flexibility (66,67). A disruption of these intricately phased patterns due to transposon integration could impact nucleosome binding positions as well as nucleosome occupancy and might provide a deeper understanding of how deviations in flexibility patterns might affect chromatin accessibility.

Clustered transposon insertion, as predicted in our polymer model, has been suggested as a major driver of the 3D organization of chromatin in a number of cellular processes. Following fertilization, in the mouse embryo, transcriptional activation of LINE-1 repeat elements regulates global chromatin accessibility (12). The compartmentalization into A and B compartments, corresponding largely to euchromatin and heterochromatin, is driven by homotypic affinities among LINE-1 and among Alu repeats, respectively (25,68). In cotton, amplification of LTR transposons increased the A compartment fraction (69). Links between transposon activation and heterochromatin decondensation upon heat stress have been reported for *Arabidopsis* (70). It was also proposed more generally that transposons contributed to the evolution of chromatin architectural features specific to humans (71). Conversely, genome compartmentalization can feed back on integration processes, as seen for retroviral integration of HIV-1 (15). Several genes that cluster in the active A compartment are recurrently targeted by HIV-1. These clustered genes, occurring proximal to superenhancers, obtain their insertion-prone localization through repositioning into the active compartment during T cell activation. Lastly, the overall unfolded state of euchromatin has been associated with the SAF-A (also called U1 snRNP or HNRNPU)-mediated retention of coding as well as noncoding RNAs (36,72,73). Many of the retained RNAs are transcribed from clustered noncoding elements (74), and our work provides a model for the emergence of such clustered placement of noncoding elements.

### Related work and limitations

While our polymer model includes changes in chromatin compaction and exclusion of transposases from compact chromatin as key mechanisms, a number of other processes and models can be considered in the explanation of clustered transposon insertion. First, regarding chromatin compaction and unfolding, several relevant biophysical mechanisms have been identified (75,76). For example, heterochromatin can be compacted by embedding in liquid-like or gel-like membrane-less organelles (77,78). Another mechanism that was suggested for heterochromatin compaction is a polymer collapse transition (79). A model that was applied to the formation of compacted chromatin clusters by the Structural Maintenance of Chromosomes proteins is bridging-induced attraction (51,80). Unfolding of euchromatin, and specifically regions harboring transcriptionally active genes as well as Alu elements, was explained via a dispersing effect of nascent and chromatin-associated RNA transcripts, leading to microphase separation from inactive chromatin (36,37,58).

Second, considering the aspect of feedback from chromatin modifications to transposon insertion, not only chromatin compaction but also other physical mechanisms have been investigated in terms of their effect on chromatin accessibility. For example, a screen of several hundred transcription factors revealed that, whereas some of the DNA-binding factors are excluded from compact chromatin, others have a strong bias to colocalize with chromatin without being affected by compaction (19). Another mechanism that might limit DNA modification to distinct domains is the formation of microphase-separated contact domains that restrict the spreading of chromatin-binding enzymes (81,82,83). Formation of such contact domains was attributed to the microphase separation of epigenetically distinct chromatin regions and was also described by block copolymer simulations (84,85,86). In addition to our findings of local changes in stiffness based on the TRX scale, previous work used polymer physics to propose that genomic loci with lower bending stiffness might favor the integration of transposons (87).

Lastly, supercoiling of the chromatin fiber was found to correlate with transcriptional activity and chromatin decompaction (88). Polymer simulations show that supercoiling can lead to clustered insertion via a positive feedback loop similar to the positive feedback we found for chromatin decompaction (89). From these different mechanisms and models, a common theme emerges: clustered insertion occurs via a positive feedback between a chromatin modification resulting from transposon insertion and further, localized insertion of transposons facilitated by this modification. Different processes can instantiate this type of feedback, implying that clustered transposon insertion similar to what we propose on the basis of our polymer model can, in fact, emerge on the basis of a number of different underlying mechanisms.

One limitation of our work is that transposases diffuse inward from the walls of the volume containing the simulated 300-kb chromatin polymer. This picture matches the “copy-and-paste” transposition mechanism. However, except for insertions occurring within this local chromatin domain, our model would be the same for cut-and-paste transposition. A polymer of 300 kb length also matches the scale at which clustered de novo insertions of LINE-1 transposons were observed (13). Chromatin domains of this size hierarchically associate into larger compartment domains (82,90), raising the question of how transposon insertion within a given 300-kb domain is related to this overall genome organization. Similar to recent findings on nonhomologous double-strand break repair, the spreading of transposon insertion might be limited by a CTCF-mediated, relative reduction of 3D chromatin-chromatin contacts between neighboring domains (83). Equally, transposon integration at a much finer scale was captured by polymer simulations with ≈10bp resolution and explicit nucleosomes, revealing a role of chromatin looping also at these length scales (91). A better understanding of how changes in chromatin accessibility might be driven by smaller-scale loop formation and proliferate into higher-order 3D interactions would require the deployment of multiscale polymer models, providing a relevant direction for future work.

Another aspect of our model that could benefit from multiscale approaches is the discrepancy between scales of transposon integration and the resulting chromatin unfolding. A single transposon can only span sequence lengths from a few hundred to a few thousand basepairs. Our bioinformatic analyses, however, indicate that regions enriched in accessible sites span up to tens of kilobases and contain several repeating transposon sequences. Our model explains this large-scale clustering and unfolding using a fixed monomer resolution of 3 kb, thereby disregarding the specific sequence length of single transposons in favor of generic monomers. Furthermore, we implement all-or-none affinity changes for these monomers, with the goal of generically representing chromatin modifications that span multiple nucleosomes. Here, multiscale approaches that realistically capture the impact of single-transposon integration events and connect them to the unfolding at higher length scales are a promising future avenue to increase physical and biological realism.

An uncontrolled sequence of transpositions, called transposon invasion, can severely disrupt genome integrity. Metazoans and plants have developed mechanisms to protect against transposon invasions, such as the piRNA pathway (92,93,94) or specific siRNAs (95). Our work suggests that certain modes of transposon insertion that lead to gradual unfolding of chromatin could make the affected chromatin region vulnerable to further invasion. Perhaps surprisingly, small transposases, which lead to sharp unfolding, could in fact be less effective in an invasion, as their unbiased insertion reduces chromatin unfolding during the initial transposition events. Transposon insertion and removal processes span a wide range of timescales, starting from the physiological timescale, on which transposon insertion and transposon silencing occur, via the intergenerational timescale of transposon silencing, up to evolutionary timescales of transposon removal or proliferation (25,96). We addressed these aspects to a limited extent by relating TRX scale-based flexibility to evolutionary age of transposons. A full investigation of the dynamics of transposon invasion, including parameter regimes or situations which offer protection against invasion, will require models that consider transposon insertion as well as transposon silencing or removal on various timescales.

## Acknowledgments

This work was supported by the Helmholtz Program “Natural, Artificial, and Cognitive Information Processing”. The study was started by discussions with Changjing Zhuge and Jinzhi Lei during an exchange visit supported by the Sino-German Center for Research Promotion. Davide Michieletto advised on the implementation of conversion reactions in LAMMPS simulations and commented on the manuscript.

## Author contributions

Study design, R.P., A.G., and L.H.; investigation, R.P. and A.G.; manuscript draft, R.P. and L.H.; manuscript editing, R.P., A.G., and L.H.

## Declaration of interests

The authors declare no competing interests.

## Supporting material

Document S1. Supplemental methods

Table S1. TRX, overlap, and cluster analysis results

Document S2. Article plus supporting material

## Footnotes

Supporting material can be found online at https://doi.org/10.1016/j.bpj.2025.03.038.
