# Supplementary material for: Chromatin unfolding via loops can drive clustered transposon insertion
Source: Biophys J. 2025 Apr 4;124(22):3908–22. doi: 10.1016/j.bpj.2025.03.038 (PMC12709442; doi:10.1016/j.bpj.2025.03.038)
Supplement: Document S1. Supplemental methods [file mmc1.pdf]

Biophysical Journal, Volume *124*

Supplemental information

Chromatin unfolding via loops can drive clustered transposon insertion

Roshan Prizak, Aaron Gadzekpo, and Lennart Hilbert

Supplemental Information

Chromatin unfolding via loops can drive clustered transposon insertion

Roshan Prizak[1,*], Aaron Gadzekpo[1,*], and Lennart Hilbert[1,2]

[1]Karlsruhe Institute of Technology, Institute of Biological and Chemical Systems, Eggenstein-Leopoldshafen, Germany
[2]Karlsruhe Institute of Technology, Zoological Institute, Karlsruhe, Germany
[*]These authors contributed equally
[*]Correspondence: lennart.hilbert@kit.edu

DETAILED MODEL DESCRIPTION

All atoms in the system (monomers and transposases) interact with phenomenological force fields (potentials) and follow Netwon's laws of motion. The following Langevin equation describes the spatial dynamics of the ith atom:

$$m_i \frac{\mathrm{d}^2\mathbf{r}_i}{\mathrm{d}t^2} = -\nabla U_i - \gamma_i \frac{\mathrm{d}\mathbf{r}_i}{\mathrm{d}t} + \sqrt{2k_\mathrm{B}T\gamma_i}\eta_i(t), \tag{1}$$

where $\mathbf{r}_i$ is the position of ith atom, m_i its mass, γ_i the friction on it from the solvent, U_i the potential experienced by it, and η_i is random uncorrelated noise with zero mean. A standard Velocity-Verlet algorithm is used to solve Eq. 1 in LAMMPS. The potential U_i is formed by accounting for all interactions between the ith atom and all other atoms. The chromatin polymer is made up of N_{tot} monomer sub-units, formed by harmonic springs between every pair, i and $i+1$, of adjacent monomers with $K_{\text{harmonic}} = 90k_\mathrm{B}T$ as:

$$U_{\text{harmonic}}(r_{i,i+1}) = K_{\text{harmonic}}(r_{i,i+1} - R_0)^2, \tag{2}$$

where $r_{i,i+1} = ||\mathbf{r}_i - \mathbf{r}_{i+1}||$ is the distance between adjacent monomers. We also consider a Kratky-Porod potential to model the bending rigidity of the polymer

$$U_{\text{BEND}}(\theta) = K_{\text{BEND}}[1 - \cos\theta], \tag{3}$$

where θ is the angle between every three adjacent monomers, and K_{BEND} is the bending energy. Persistence length is given by $L_p = K_{\text{BEND}}\sigma$. Repulsion between atoms is modeled as a Weeks-Chandler-Andersen (WCA) potential, which is a shifted and truncated version of a Lennard-Jones (LJ) potential. The WCA potential for a pair of atoms i and j is

$$U_{\text{WCA}}(r_{ij}) = \begin{cases} 4\left[\left(\frac{d_{ij}}{r_{ij}}\right)^{12} - \left(\frac{d_{ij}}{r_{ij}}\right)^{6}\right] + 1, \text{if } r_{ij} < 2^{\frac{1}{6}} d_{ij} \\ 0, \text{otherwise}, \end{cases} \tag{4}$$

with $r_{i,i+1} = ||\mathbf{r}_i - \mathbf{r}_{i+1}||$ is the distance between the atoms, and d_{ij} is usually the mean diameter of the atoms i and j (See below for details on size-based exclusion).

Monomers come from one of two types, native and transposed. Native monomers attract each other with an interaction energy E, while transposed monomers do not exhibit attraction. Native monomers represent the default chromatin monomer type while transposed monomers represent those with a transposon insertion and hence do not exhibit attraction. These attractive interactions are modeled as shifted, truncated Lennard-Jones potential

$$U_{\text{LJcut}} = \begin{cases} U_{\text{LJ}_0}(r_{ij}) - U_{\text{LJ}_0}(r_{\text{cut}}), \text{if } r_{ij} < r_{\text{cut}} \\ 0, \text{otherwise}, \end{cases} \tag{5}$$

where the Lennard-Jones potential $U_{\text{LJ}_0}(r)$ is

$$U_{\text{LJ0}}(r) = 4E\left[\left(\frac{d_{ij}}{r}\right)^{12} - \left(\frac{d_{ij}}{r}\right)^{6}\right], \tag{6}$$

and the cut-off distance is given by $r_{\text{cut}} = 2.5\sigma$. If native monomers are within a distance between $2^{1/6}\sigma$ and r_{cut}, they feel an attraction.

Simulation parameters

In the chromatin polymer, each monomer corresponds to a chromatin segment of size 3 kbp and has a diameter $\sigma = 30$ nm, assuming a compaction of around 100 bp/nm. This value compares well with a previous estimate of 90-130 bp/nm (1). We consider $K_{\text{BEND}} = 3\ k_{\text{B}}T$, resulting in $L_p = 3\sigma = 90$ nm. The persistence length of chromatin is not understood well, but experimentally observed values range between 50-200 nm (2) or 66–134 nm (3), corresponding favorably to our parameter value.

We consider a default interaction energy of $E = 1.5\ k_{\text{B}}T$, in line with parameters used in previous chromatin polymer simulations (2, 4). Each run of a polymer simulation consists of 1 million timesteps, with each timestep of length $\mathrm{d}t = 0.01\tau$, where $\tau = \sqrt{\sigma^2 m / k_{\text{B}}T}$ is the natural timescale of the system. All components of the system are assumed to have the same mass ($m = 1$).

In the transposase model, we have 25 transposase molecules that can mediate transposon insertion. We vary the effective transposase size $s_T\sigma$ between $0.2\sigma = 6$ nm and $0.9\sigma = 27$ nm, and the insertion rate over two orders of magnitude $k_T \in [0.1, 10]$, corresponding to a fastest rate of an insertion attempt every 100 timesteps and a slowest rate of an insertion attempt every 10000 timesteps.

Simulation steps

Each simulation run consists of three phases - an initial “soft” phase, an equilibration phase, and then the main simulation phase.

Insertion process

The chromatin polymer with a total of N_{tot} monomers and N_{tr} transposons inside it can be represented as s, a binary string of length N_{tot} with 0’s representing native monomers and 1’s representing transposed monomers. As the chromatin polymer is joined at the ends, any shifted version of s is equivalent to s. We call this the polymer state. For example, if $N_{\text{tot}} = 6$, a possible polymer state with two transposons is $s(2) = 011000$. This is equivalent to its shifted versions 110000, 100001, 000011, 000110 and 001100.

A transposon insertion event consists of selecting one of the $N_{\text{tot}} - N_{\text{tr}}$ 0’s from the current polymer state $s(2)$ and replacing it with a 1 to obtain a new polymer state $s(3)$. For example, an insertion in the first position of $s(2) = \mathbf{0}11000$ would lead to $s(3) = 111000$. However, an insertion could have occurred in one of the other three available positions (0’s), giving rise to $s(3) = 011\mathbf{1}00, 0110\mathbf{1}0$ or $01100\mathbf{1}$.

How are these insertion positions selected? Given a polymer state, each monomer m_i at the ith position has two adjacent neighbours, usually at the $i-1$ and $i+1$ positions. The first and the last monomer in any given s are neighbours of each other. Insertion can be of two types - random (R) or adjacent (A). Random insertion involves insertion into one of the available native monomers (0). Adjacent insertion involves insertion into a native monomer (0) with at least one transposon (1) as one of its two neighbours.

In the basic model, each insertion’s type is first chosen from the two options - adjacent insertion type with probability α (placement adjacency), or a random insertion type with probability $1 - \alpha$. If the chosen insertion type is adjacent, one of the available native monomers (0) which has a transposon neighbour (1) is chosen. The local substring in the polymer state around the chosen position would be one of the following three possibilities – (..1**0**0..), (..0**0**1..), or (..1**0**1..). If the chosen insertion type is random, one of the $N_{\text{tot}} - N_{\text{tr}}$ available native monomers (0) is picked randomly. The local substring in the polymer state around the chosen position would be one of the following four possibilities – (..0**0**0..), (..1**0**0..), (..0**0**1..), or (..1**0**1..). Note that even though the chosen insertion type is random, three of the possible chosen positions have a transposon neighbour, and hence a random insertion can also result in a seemingly adjacent insertion.

In the transposases model, insertion positions are selected based the proximity between native monomers and transposases. In an insertion event, the set of native monomers with a transposase within a distance of $(1 + s_T)\sigma$ from each of their centers are selected and converted to a transposon. Unlike the basic model, the insertion positions do not depend on a pre-specified placement adjacency.

Insertion history

In a single simulation run, starting from a polymer with no transposons $s(0) = 000\ldots000$, transposons are sequentially inserted at various positions, with the polymer state changing with each insertion. Each insertion changes one of the 0's in the polymer state to a 1. The simulation ends when all monomers are transposed $s(N_{\mathrm{tot}}) = 111\ldots111$. The set of polymer states $s(i), i = 0, 1, \ldots N_{\mathrm{tot}}$ together is called the insertion history.

Hill curve fit

We fit a Hill curve to $R_{\mathrm{g}}(N_{\mathrm{tr}})$ to obtain the Hill exponent n and the half-point K:

$$f(x) = R_{\mathrm{g}}(0) + (R_{\mathrm{g}}(N_{\mathrm{tot}}) - R_{\mathrm{g}}(0))(1 + K^n)\frac{x^n}{x^n + K^n}, \tag{7}$$

where $x = N_{\mathrm{tr}}/N_{\mathrm{tot}}$ is the fraction of transposons.

Estimation of α

The insertion history in the transposase model emerges from the interactions between transposases and chromatin polymer, and the accessibility constraints resulting from transposase size and insertion rate. This is in contrast with the basic model without transposases, in which insertion history was generated from a single parameter α, the placement adjacency. To compare the insertion histories between the basic model and the transposase model, we estimate the underlying α in the transposase model using a maximum likelihood estimation (MLE) method on the set of resulting histories. This MLE estimate α_{mle} can be interpreted as the most likely placement adjacency value α that would generate the resulting set of insertion histories if the underlying model were the basic model.

Given an insertion history $s(i), i = 0, 1, \ldots, N_{\mathrm{tot}}$, for each insertion $s(i) \to s(i+1)$, in $s(i)$ we check if the inserted position has a transposon neighbour (..1**0**0..), (..0**0**1..), or (..1**0**1..) - say, event A, or if neither of the two neighbours are transposons (..0**0**0..) - say, event B. We also note down the total number of native monomers in $s(i)$ that have a transposon neighbour – m. So the number of native monomers in $s(i)$ that do not have a transposon neighbour is $N_{\mathrm{tot}} - i - m$. We define

$$p_i = \frac{m}{N_{\mathrm{tot}} - i}. \tag{8}$$

We suppose the resulting insertion history is generated with an underlying placement adjacency α. For each insertion, with probability α, insertion occurs in a position that has an adjacent transposon (event A), and with probability $1 - \alpha$, the insertion position is chosen randomly. However, one of the randomly chosen positions can have an adjacent transposon (probability p_i), and hence result in an event A. With probability $1 - p_i$, an event B can occur. Hence, the overall probability of event A occurring is $\alpha + (1 - \alpha)p_i$, and the probability of event B occurring is $(1 - \alpha)(1 - p_i)$. Each insertion in the insertion history can be classified as either an event A or an event B, leading to a string of A's and B's of length N_{tot}. From this, we can write the expression for the log likelihood of the first T insertions of the given insertion history as

$$l(\alpha) = \sum_{i=1}^{T} \log Q_i, \tag{9}$$

where $Q_i(\alpha) = \alpha + (1 - \alpha)p_i$ if the ith insertion is an event A, and $Q_i(\alpha) = \alpha + (1 - \alpha)p_i$ if the ith insertion is an event B. Now, if r individual repeats of the simulation are performed, we have r simulation histories $s_j(i), i = 0, 1, \ldots, N_{\mathrm{tot}}, j = 1, 2, \ldots, r$. We can write the log likelihood of the r insertion histories as

$$l(\alpha) = \frac{1}{r}\sum_{j=1}^{r}\sum_{i=1}^{T} \log Q_{ij}, \tag{10}$$

where $Q_{ij}(\alpha) = \alpha + (1 - \alpha)p_{ij}$ if the ith insertion of the jth run is an event A, and $Q_{ij}(\alpha) = \alpha + (1 - \alpha)p_{ij}$ if the ith insertion of the jth run is an event B. As the likelihood L is a function of the placement adjacency α, we can find the maximum likelihood estimate of α as

$$\alpha_{\mathrm{mle}} = \underset{\alpha}{\operatorname{argmax}}\; l(\alpha), \tag{11}$$

which is the value of α that maximizes the likelihood of obtaining the given r insertion histories.

REFERENCES

1. Bajpai, G., and R. Padinhateeri, 2020. Irregular Chromatin: Packing Density, Fiber Width, and Occurrence of Heterogeneous Clusters. *Biophysical Journal* 118:207–218. https://doi.org/10.1016/j.bpj.2019.11.004.

2. Buckle, A., C. A. Brackley, S. Boyle, D. Marenduzzo, and N. Gilbert, 2018. Polymer simulations of heteromorphic chromatin predict the 3D folding of complex genomic loci. *Molecular Cell* 72:786–797. https://doi.org/10.1016/j.molcel.2018.09.016.

3. Dekker, J., 2008. Mapping in vivo chromatin interactions in yeast suggests an extended chromatin fiber with regional variation in compaction. *Journal of Biological Chemistry* 283:34532–34540. http://dx.doi.org/10.1074/jbc.M806479200.

4. Brackley, C. A., S. Taylor, A. Papantonis, P. R. Cook, and D. Marenduzzo, 2013. Nonspecific bridging-induced attraction drives clustering of DNA-binding proteins and genome organization. *Proceedings of the National Academy of Sciences USA* 110:E3605–E3611. http://www.pubmedcentral.nih.gov/articlerender.fcgi?artid=3780866&tool=pmcentrez&rendertype=abstract.

5. Giordano, J., Y. Ge, Y. Gelfand, G. Abrusán, G. Benson, and P. E. Warburton, 2007. Evolutionary history of mammalian transposons determined by genome-wide defragmentation. *PLOS Computational Biology* 3:e137. https://doi.org/10.1371/journal.pcbi.0030137.

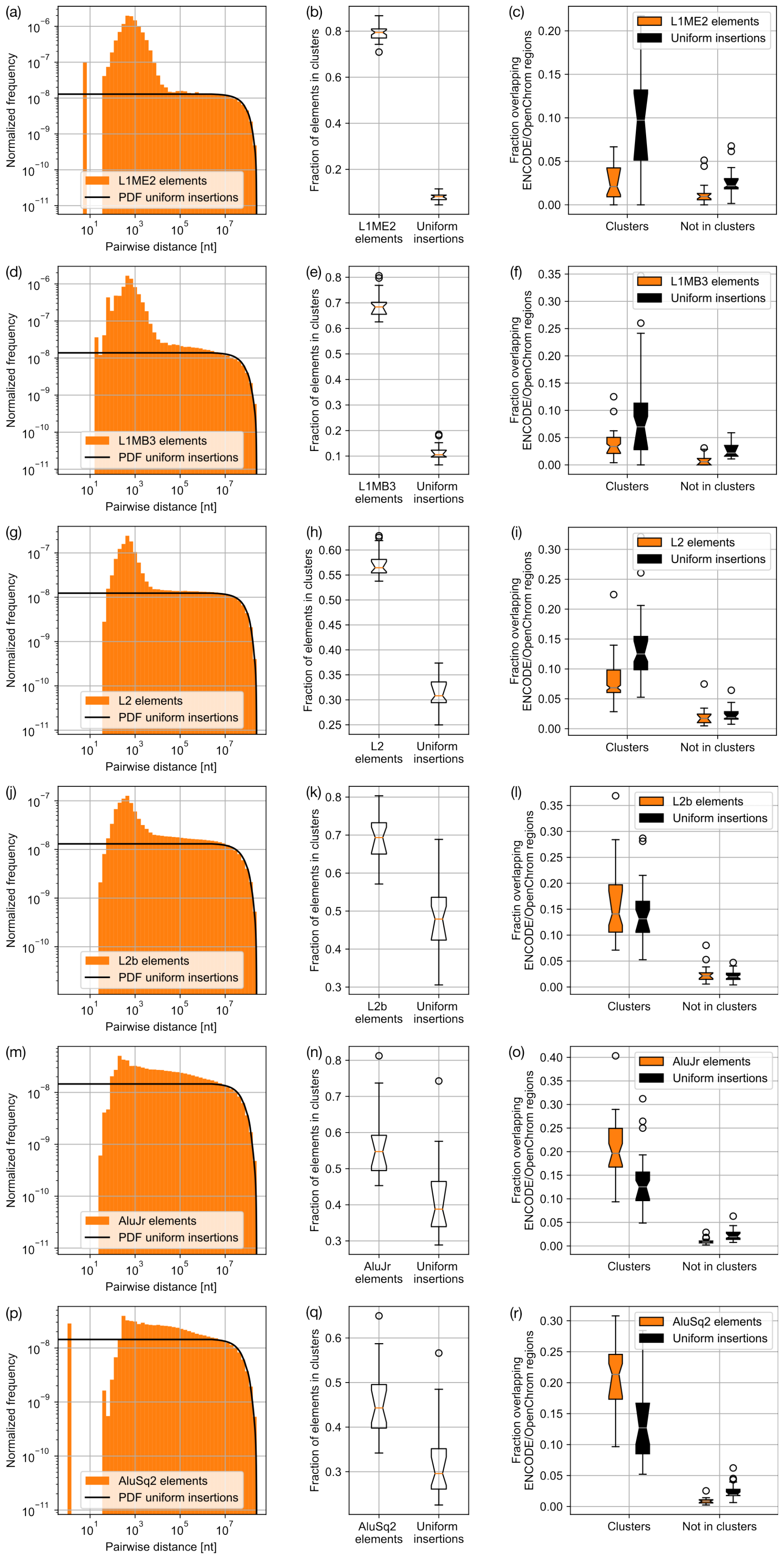

Figure 1: **Clustering analysis of additional Alu and LINE transposons** The first column shows normalized histograms of pairwise transposon distances compared to the probability density of uniformly distributed insertions. The second column shows the fraction of transposons or uniformly distributed insertions that are part of clusters detected by DBSCAN (max. dist.= 10,000 Nt, min. points=2). The third column shows the fraction of clusters or elements that are not part of a cluster, that overlap open chromatin. All transposons show an increase in short pairwise distances, which is very pronounced for LINE elements and more dispersed for Alu elements. In line with a higher frequency of short pairwise distances, a higher fraction of LINE elements are found in clusters. However, both LINE and Alu elements are found more frequently in clusters than expected from uniformly distributed elements. When compared to uniformly distributed elements of the same length, overlap with open chromatin ranges from reduced (L1ME2) to only weakly reduced (L1MB3, L2) to comparable (L2b). For AluJr and AluSq2, an increased overlap with open chromatin is found.

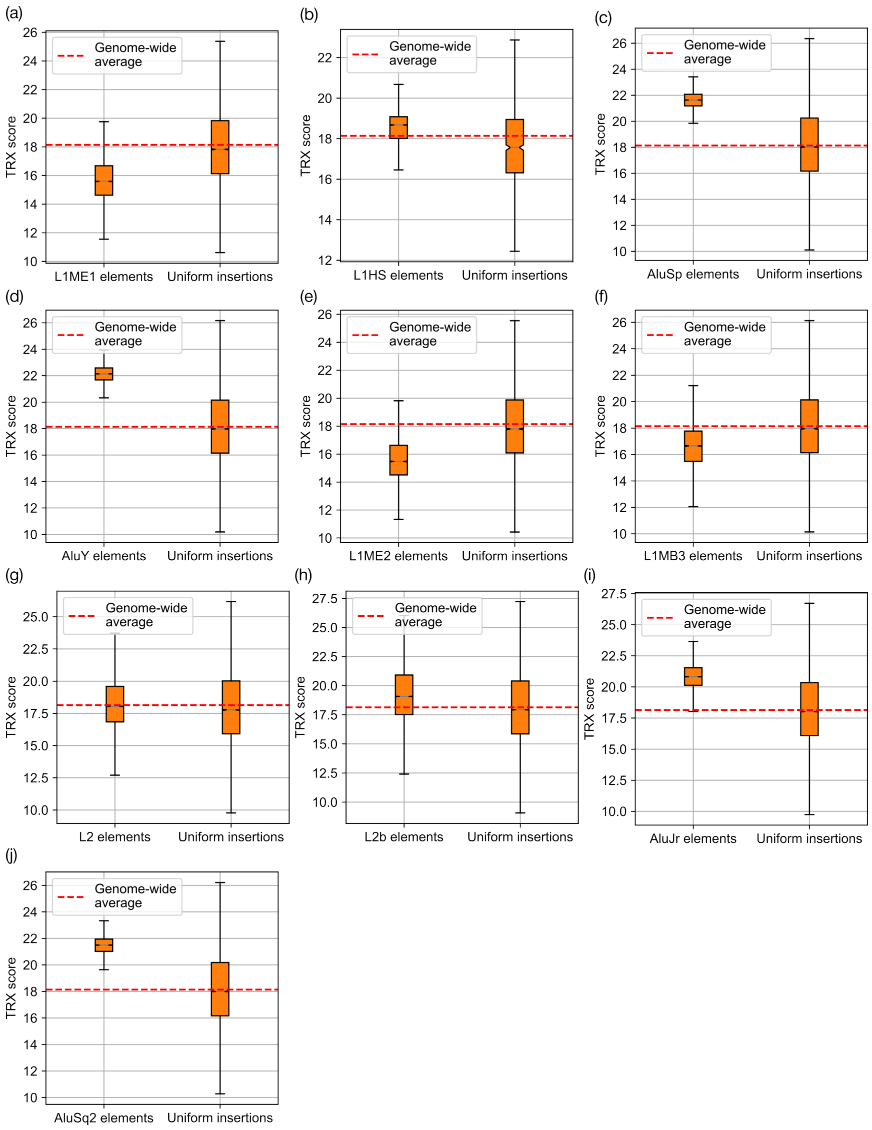

Figure 2: **TRX scores of transposons and uniformly distributed reference elements** TRX scores for a selection of transposons and uniformly distributed reference elements of the same lengths. Boxplots contain TRX scores for all elements belonging to one subfamily (or corresponding reference elements). Older L1 subfamilies (L1ME1, L1ME2, L1MB3) show reduced TRX scores compared to the reference, newer L1 subfamilies (L1HS) and L2 elements are at or slightly above the genome-wide average, and Alu subfamilies show strongly increased scores. Boxplots contain thousands of TRX scores resulting in low P values ($P<10^{-57}$) when comparing transposons and reference elements.

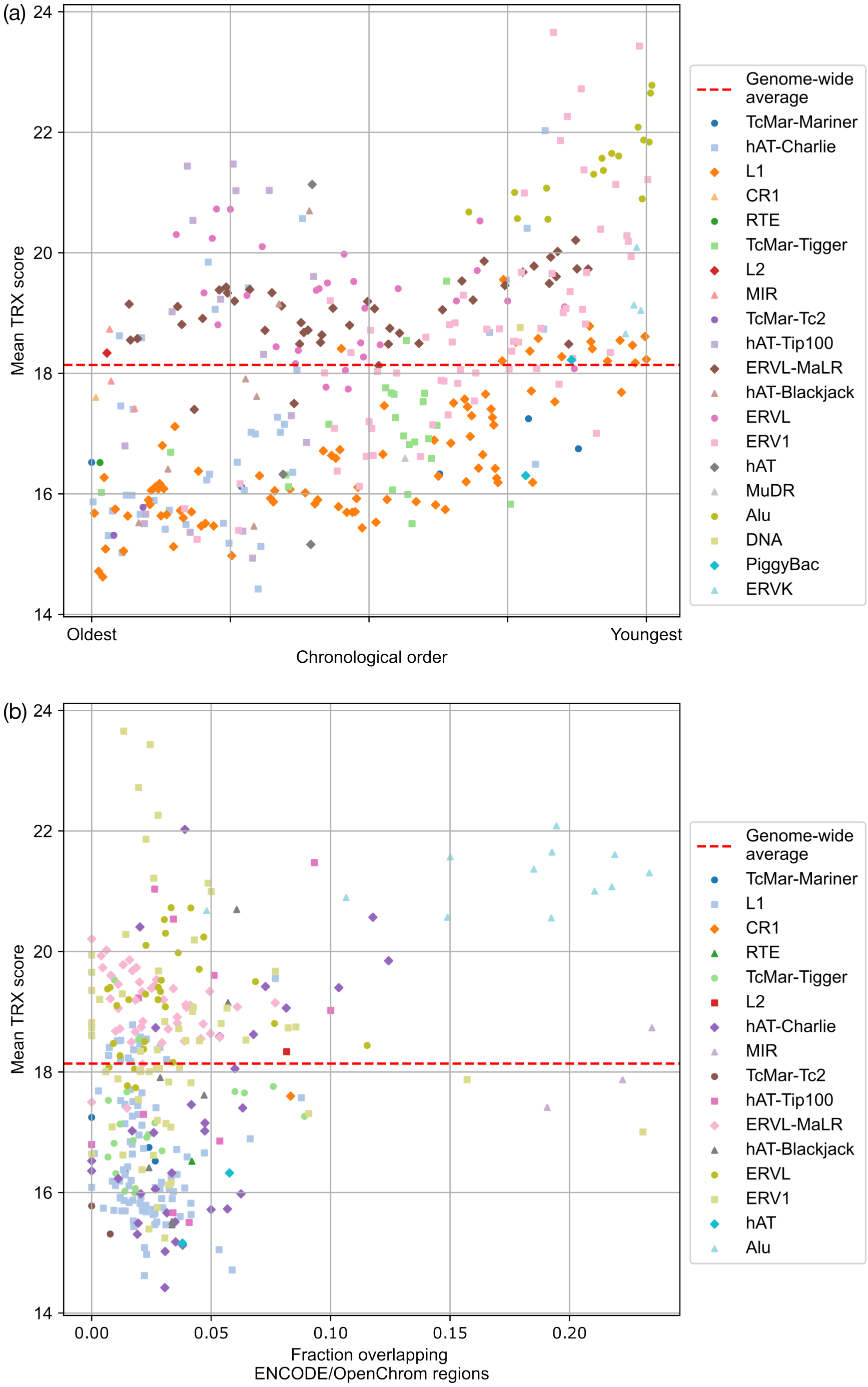

Figure 3: **TRX scores against transposon age and overlap with open chromatin** (a) The chronological ordering of transposons was based on the level of defragmentation (5). Each datapoint represents the averaged TRX score of all members of a transposon subfamily, such as L1ME1. Subfamilies belonging to one family are labelled on the right. Younger transposon subfamilies tend to have higher TRX scores. This trend is well visible for some subfamilies such as Alu, L1 and ERV1 but seems to be absent in others such as hAT-Tip100. 360 subfamilies matched with the hg19 repeat masker annotation were used in total. (b) TRX scores for transposon subfamilies plotted against overlap of clusters with open chromatin. Cluster detection was carried out with DBSCAN (max. dist.= 10,000 Nt, min. points=2). 321 subfamilies that have at least ten clusters on a least one chromosome were included. The Alu and L1 families stand out by predominantly occupying the upper right and lower left corner respectively.
